# Primary ILM peeling during retinal detachment repair: a systematic review and meta-analysis

**DOI:** 10.1038/s41598-023-30060-w

**Published:** 2023-03-03

**Authors:** David Lamas-Francis, Manuel Bande-Rodríguez, María José Blanco-Teijeiro

**Affiliations:** 1 Department of Ophthalmology, Hospital de Conxo, University Hospital of Santiago de Compostela, Ramón Baltar s/n, 15706 Santiago de Compostela, Spain; 2 Department of Surgery, University of Santiago de Compostela, Santiago de Compostela, Spain

**Keywords:** Outcomes research, Surgery

## Abstract

Epiretinal membrane (ERM) formation is a known postoperative complication following retinal detachment (RD) repair surgery. Prophylactic peeling of the internal limiting membrane (ILM) during surgery has been shown to reduce the risk of developing postoperative ERM formation. Some baseline characteristics and degrees of surgical complexity may act as risk factors for ERM development. In this review we aimed to investigate the benefit of ILM peeling in patients without significant proliferative vitreoretinopathy (PVR) who underwent pars plana vitrectomy for RD repair. A literature search using PubMed and various keywords retrieved relevant papers from which data were extracted and analyzed. Finally, the results of 12 observational studies (3420 eyes) were summarized. ILM peeling significantly reduced the risk of postoperative ERM formation (RR = 0.12, 95% CI 0.05–0.28). The groups did not differ in final visual acuity (SMD 0.14 logMAR (95% CI − 0.03–0.31)). The risk of RD recurrence (RR = 0.51, 95% CI 0.28–0.94) and the need for secondary ERM surgery (RR = 0.05, 95% CI 0.02–0.17) were also higher in the non-ILM peeling groups. In summary, although prophylactic ILM peeling appears to reduce the rate of postoperative ERM, this benefit does not translate into consistent visual recovery across studies and potential complications must be considered.

## Introduction

Macular epiretinal membrane (ERM) development is one of the most common postoperative complications of pars plana vitrectomy (PPV) for retinal detachment (RD) repair. The reported incidence of postoperative ERM formation following PPV is variable (6–70%)^1–13^, depending on the study design and complexity of the eyes reviewed. ERM formation may be under-reported in older studies in which postoperative macular OCT scans were not performed. A recent meta-analysis including data on 2380 eyes that had undergone PPV for retinal detachment and reported a 7.10% rate of postoperative ERM formation, which is comparable to the rate associated with repair by scleral buckling alone (5.27%)^14^. Postoperative visual acuity is often limited by ERM formation^1,15^, and a secondary ILM peeling surgery may be necessary to restore vision.

In order to increase its visibility, the ILM is usually stained with vital dyes such as indocyanine green, trypan blue, brilliant blue G or triamcinolone. Although the use of these dyes may reduce mechanical trauma, some studies have shown that they may be toxic to the neurosensory retina and the retinal pigment epithelium^16^. ILM peeling can increase central macular thickness, cause distortion of retinal layers and induce scotoma formation^17^. Long-term studies on these side effects are needed to assess the extent of the damage and its functional repercussion.

Risk factors for postoperative ERM formation are long axial length, the use of 20G (rather than 23 or 25G) ports, use of silicone oil, high photocoagulation energy, type 2 diabetes and preoperative proliferative vitreoretinopathy (PVR)^15,18^. The benefit of ILM peeling should be evaluated by taking into account the varying degrees of surgical complexity and different surgical techniques as well as differences in preoperative macular status (on/off; preoperative ERM) and the rate of PVR. Other review studies have demonstrated the advantages of prophylactic ILM peeling during VPP for RD repair^19,20^, although these included patients with various degrees of PVR which determined the type of surgical repair used.

In view of these limitations, we conducted a systematic review and meta-analysis to examine the effectiveness and safety of prophylactic ILM peeling during primary RD repair in eyes without significant PVR.

## Results

### Literature search and study characteristics

**Figure 1:**
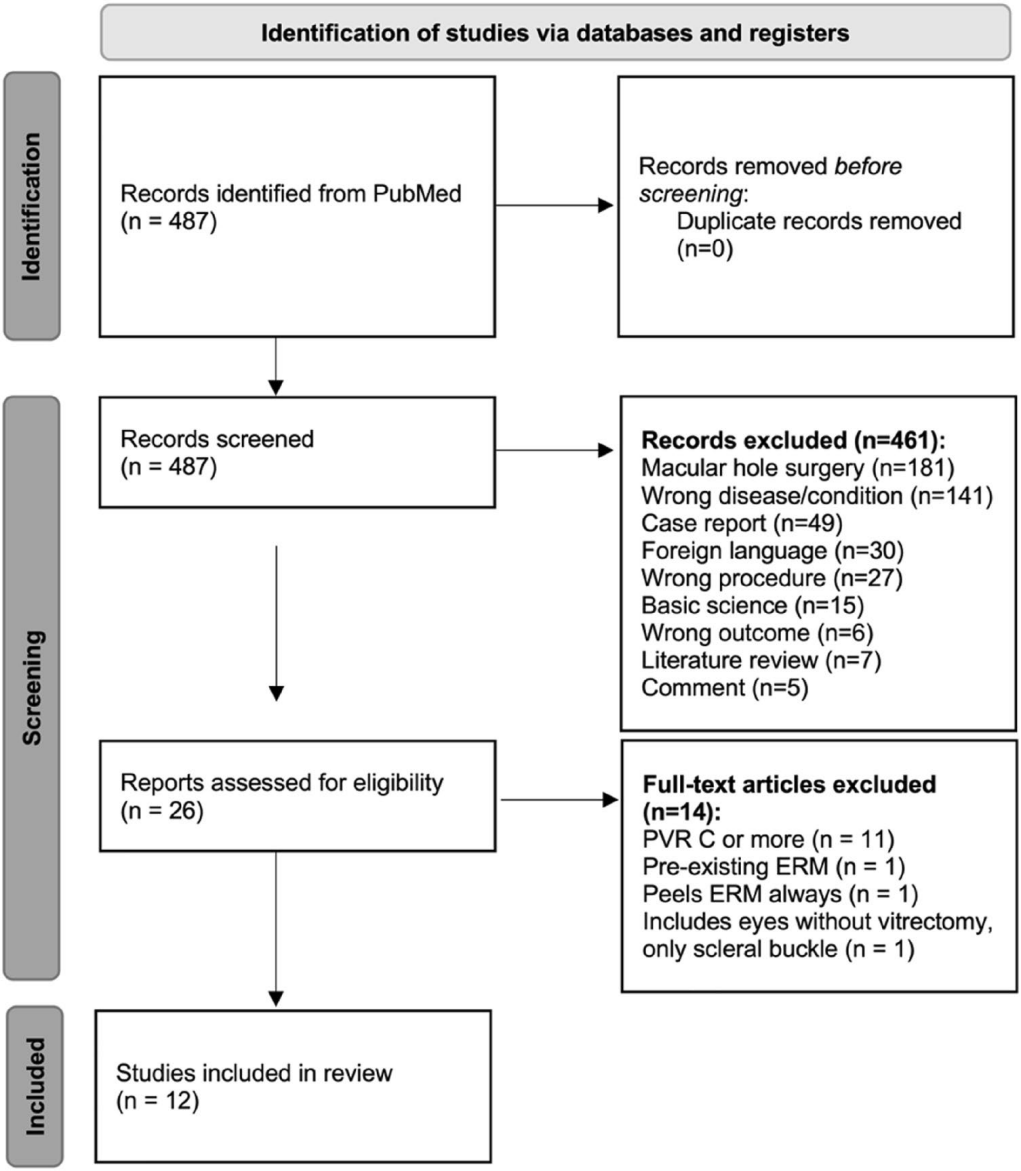
PRISMA flow diagram for study selection.

The initial database search conducted on September 9, 2022 retrieved a total of 487 titles (see Fig. 1 for the PRISMA flow diagram). No duplicates were detected. The 487 titles and abstracts were reviewed, and 461 records were excluded as they did not meet the eligibility criteria. The full-text version of the remaining 26 articles were assessed, and 14 were excluded. A total of 12 studies were finally included in this review.

A total of 10 retrospective case series and two prospective non-randomized studies were included in the meta-analysis. Overall, 3420 eyes were included: 731 in the ILM peeling group and 2661 in the non-ILM peeling group. The main characteristics of the studies reviewed are summarized in Table 1.

**Table 1:** Summary of the main characteristics of the studies included in the review.

	Study design	Preoperative macula status	PVR	Total eyes	Min. Follow-up (months)	Port gauge	ILM stain	Tamponade gas	Combined procedures
Rao et al. 2013	Retrospective cohort	On/off P 25%/75% NP 27%/63%	No PVR	62	6	ns	Triamcinolone P 100%	ns	Scleral buckle P 80%, NP 91%
Nam et al. 2015	Retrospective cohort	On/off P 59%/41% NP 54%/46%	No PVR	135	12	20G	ICG P 100%	SF6/C3F8 P 14%/86%, NP 89%/11%	Cataract*
Akiyama et al. 2016	Retrospective cohort	On/off P 64%/36% NP 50%/50%	No PVR	102	6	23 or 25G	Triamcinolone P 97%, NP 52%	air/SF6/C3F8/SO*	Cataract/scleral buckle*
Foveau et al. 2018	Retrospective cohort	Off	PVR B	75	6	23G	Brilliant blue P 100%	SF6 100%	Cataract P 24%, NP 32%
Blanco-Teijeiro et al. 2018	Retrospective cohort	Off	No PVR	62	12	23G	Brilliant blue P 100%	SF6 100%	Scleral buckle*
Akiyama et al. 2018	Retrospective cohort	On	No PVR	55	6	23 or 25G	Triamcinolone*	ns	–
Ishida et al. 2019	Retrospective cohort	On/off*	PVR B or lower	322	12	25G	Brilliant blue P 100%	Air/SF6/C3F8*	Cataract*
Abdullah et al. 2020	Prospective non-randomized	Off	PVR A or lower	60	6	23G	Brilliant blue P 100%	SO 100%	Cataract P 0%, NP 10%
Arias et al. 2020	Retrospective cohort	On/off P 30%/70% NP 36%/64%	PVR B or lower	140	6	23G	MB Dual 100%	SF6/C3F8 P 74%/13% NP 70%/21%	Cataract/scleral buckle P 17%/30% NP 61%/24%
Starr et al. 2020	Retrospective cohort	On/off P 41%/59% NP 51%/49%	No PVR	1442	6	ns	ns	ns	Scleral buckle P 49%, NP 39%
Obata et al. 2021	Retrospective cohort	On/off P 60%/40% NP *	No PVR	887	6	ns	Triamcinolone/brilliant blue/ICG/trypan blue P 85%/62%/11%/0% NP 84%/1%/0%/1%	Air/SF6/C3F8/SO P 16%/81%/0%/2% NP 24%/73%/1%/2%	Cataract/scleral buckle P 93%/1% NP 81%/5%
Akiyama et al. 2021	Prospective non-randomized	On/off P 68%/32% NP 32%/68%	PVR B or lower	78	6	23 or 25G	Triamcinolone/brilliant blue*	SF6/SO*	Cataract/scleral buckle P 77%/0% NP 71%/4%

ns, not specified; PVR, proliferative vitreoretinopathy; Min., minimum; ILM, internal limiting membrane; ICG, indocyanine green; SF6, sulphur hexafluoride; C3F8, perfluoroctane; SO, silicone oil. P, ILM-peeling group. NP, non ILM-peeling group.

*Percentage not specified in source study.

The risk of bias assessment determined using the Newcastle–Ottawa scale is provided in Table 2. All the studies included in this meta-analysis were considered to have low risk of bias.

**Table 2:** Newcastle–Ottawa scale for risk of bias assessment.

	Study design	Selection	Comparability	Outcomes	Total score
Rao et al. 2013	Retrospective cohort	3	1	3	7
Nam et al. 2015	Retrospective cohort	3	1	3	7
Akiyama et al. 2016	Retrospective cohort	3	1	3	7
Foveau et al. 2018	Retrospective cohort	3	1	3	7
Blanco-Teijeiro et al. 2018	Retrospective cohort	3	1	3	7
Akiyama et al. 2018	Retrospective cohort	3	1	3	7
Ishida et al. 2019	Retrospective cohort	3	2	3	8
Abdullah et al. 2020	Prospective non-randomized	3	1	2	6
Arias et al. 2020	Retrospective cohort	3	2	3	8
Starr et al. 2020	Retrospective cohort	3	1	3	7
Obata et al. 2021	Retrospective cohort	3	2	3	8
Akiyama et al. 2021	Prospective non-randomized	4	2	2	8

### Rate of macular epiretinal membrane formation

**Figure 2:**
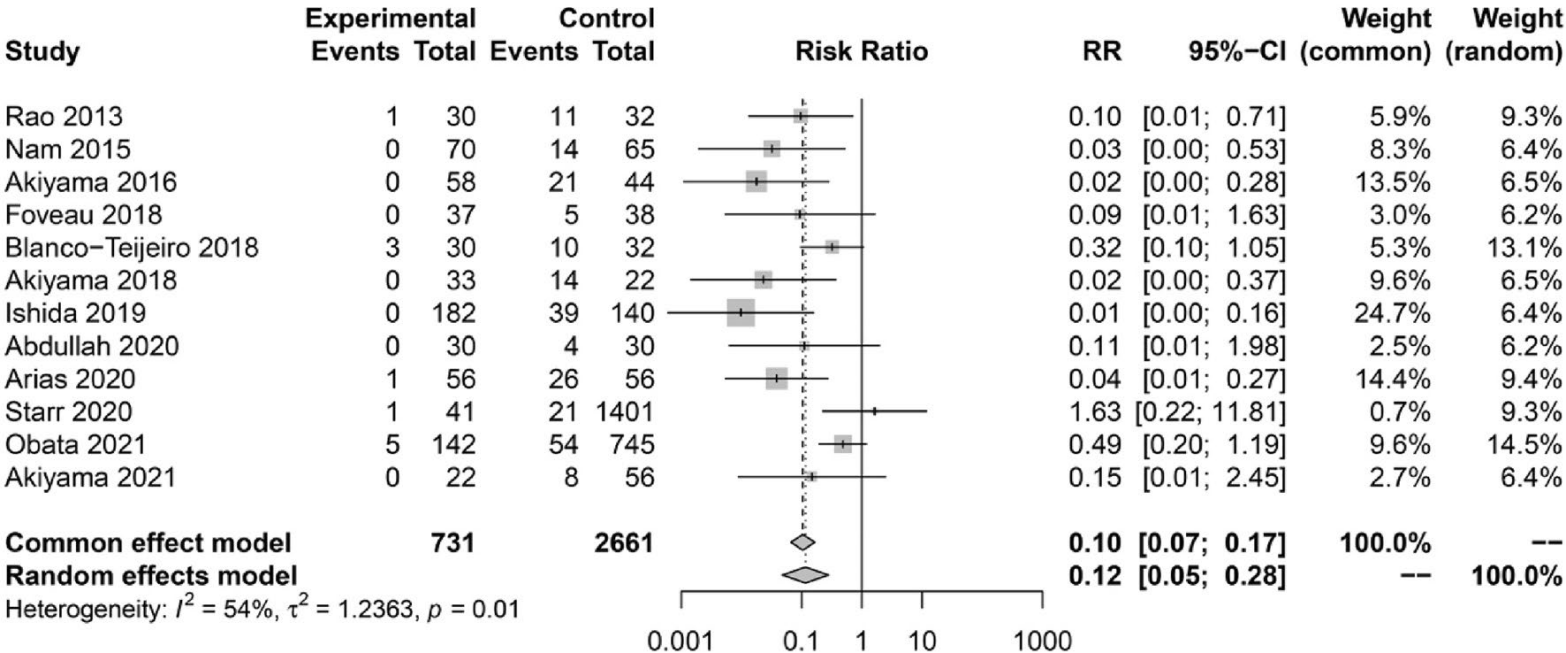
A forest plot showing the rate of epiretinal membrane formation following primary RD repair with and without prophylactic ILM peeling.

Postoperative ERM was observed in 11 (1.50%) eyes in the ILM-peeling group and in 227 (8.53%) eyes in the non-ILM peeling group. The meta-analysis showed a significantly lower rate of postoperative ERM formation in the ILM-peeling group than in the non-peeling group (risk ratio, RR = 0.12, 95% CI 0.05–0.28) (Fig. 2).

### Change in visual acuity

**Figure 3:**
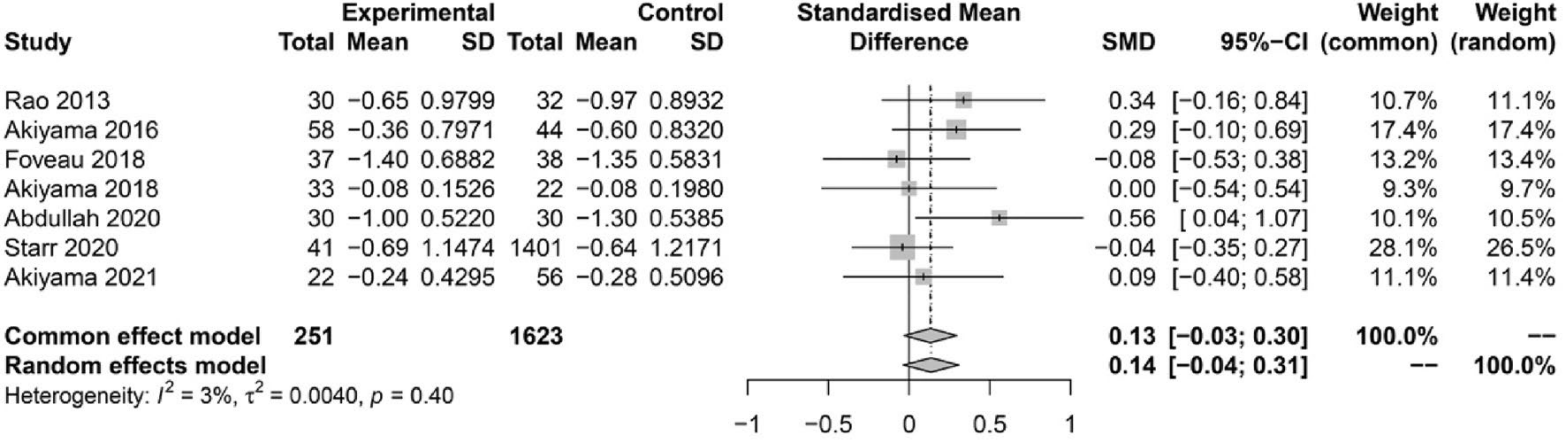
Forest plot showing the standardized mean difference (SMD) and 95% confidence intervals (CI) for BCVA change.

A total of 7 studies (1623 eyes) reported preoperative and postoperative BCVA as a mean value with SD. The standardized mean difference (SMD) was 0.14 logMAR (95% CI − 0.04–0.31), and therefore no statistical difference in mean BCVA change was detected between ILM peeling and non-ILM peeling groups (Fig. 3).

### Rate of recurrence of retinal detachment

**Figure 4:**
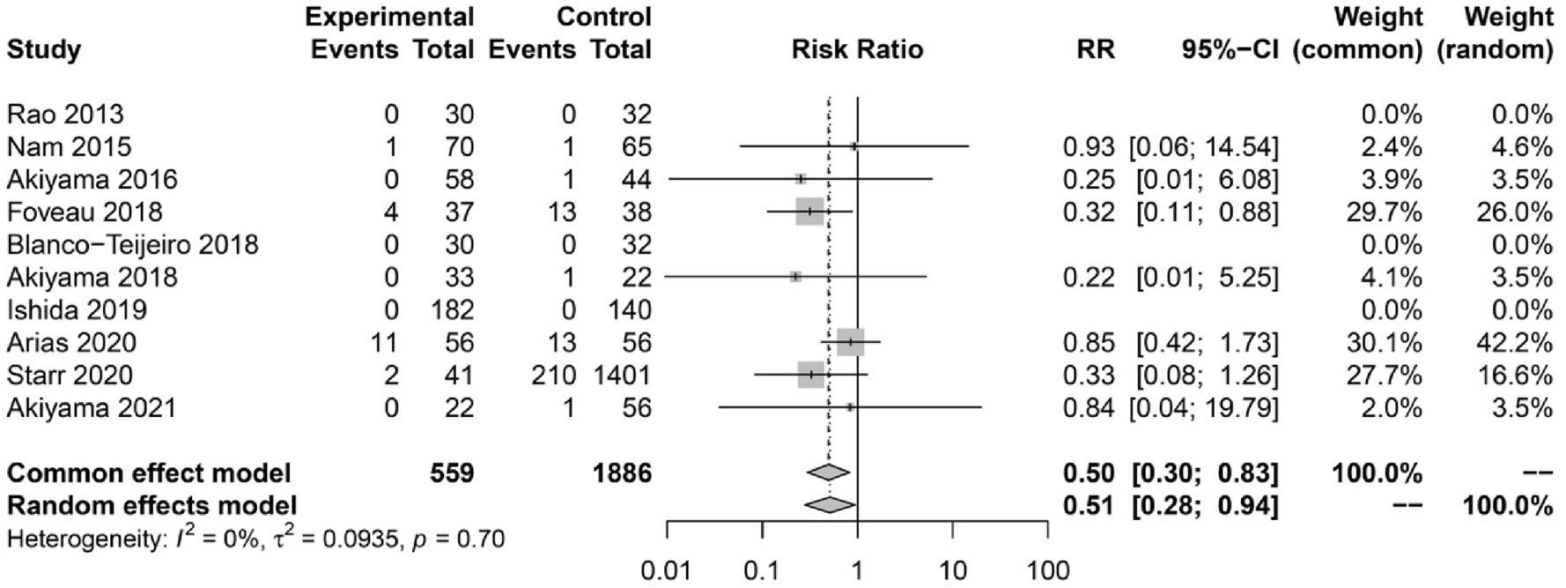
Forest plot showing the risk ratio (RR) and 95% confidence intervals (CI) comparing the rate of RD recurrence between groups.

A total of 10 studies disclosed data on RD recurrence. Overall, 12.73% (240/1886) eyes presented recurrence in the non-ILM peeling group and 3.22% (18/559) eyes in the ILM-peeling group. The meta-analysis comparing the rate of RD recurrence between groups showed a significantly higher rate in the non-ILM peeling group (RR = 0.51, 95% CI 0.28–0.94) (Fig. 4).

### Need for secondary epiretinal membrane surgery

**Figure 5:**
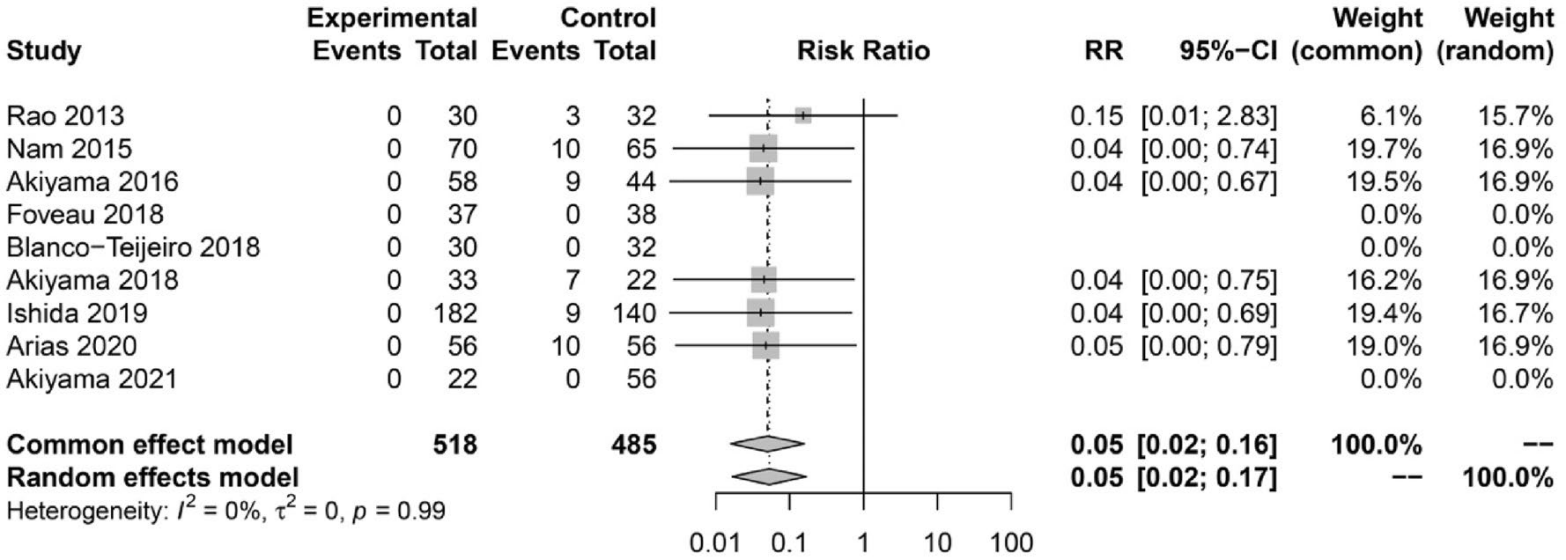
Forest plot showing the rate of secondary ERM surgery in the ILM-peeling and non-ILM peeling groups.

A total of 9 studies reported data on secondary ERM surgery. Overall, 9.90% (48/485) patients from the non-ILM peeling group required secondary ERM surgery, whilst none (0/518) of the patients who received primary ILM peeling required a secondary ERM intervention. The meta-analysis showed a significantly higher rate of secondary ERM surgery in the non-ILM peeling group compared to the ILM peeling group (RR = 0.05, 95% CI 0.02–0.17) (Fig. 5).

### Other characteristics

Meta-regression analysis demonstrated that the main outcome (rate of postoperative ERM) was not affected by different follow-up periods (6 or 12 months), trocar gauge (20G or 23-25G) or stain used during surgery (triamcinolone or brilliant blue/membrane blue). Other variables (tamponade gas, combined procedures, macular status) were not considered as data was not always stratified in the studies. Detailed results are given in Supplementary File 1.

We assessed potential risk of publication bias through visual analysis of funnel and Galbraith plots (Supplementary Files 2 and 3). Most studies provided a RR within the expected 95% CI, however two studies (by Obata et al. and Starr et al.) show no benefit with primary ILM peeling. This intervention was carried out less thoroughly in both larger studies (2.8% and 16.0% of patients) than in smaller studies (28 to 57%), which may result in smaller effect estimates. This potential source of heterogeneity may have led to some degree of asymmetry in the funnel and Galbraith plots.

## Discussion

To the best of our knowledge, this study is the most up-to-date systematic review and meta-analysis examining the effectiveness of primary ILM peeling during RD repair. The results of this meta-analysis demonstrated a significantly lower incidence of postoperative ERM in patients who underwent primary ILM peeling during RD repair surgery. A previous review by Fallico et al.^20^, which included 769 eyes from 9 studies, showed similar outcomes. Yannuzzi et al.^19^ published a cost analysis of 586 eyes across 6 studies, suggesting that primary ILM peeling may significantly reduce the rate of postoperative ERM and need for secondary surgery, thereby justifying the higher cost of primary ILM peeling. Both reviews included patients with complex cases with advanced PVR, requiring retinectomy and use of silicone oil. We decided to exclude studies involving these patients, as both advanced PVR and silicone oil are known risk factors for postoperative ERM formation^15,18^ and their inclusion could lead to overestimation of the effects of the intervention. In addition, recent studies have suggested that the current classification of PVR^21^ has important limitations: early stages (A or B) are commonly under-reported, there is a certain level of subjectivity and correct classification remains complex in some cases^22^. A new classification of PVR is deemed necessary, providing information on intraretinal changes, progressiveness and allowing appropriate treatment guidance^23^.

Of the total of 12 studies (3420 eyes) included in this study, two were non-randomized prospective studies and 10 were retrospective case series. Based on the meta-analysis of the data from these studies we demonstrated that prophylactic ILM peeling during RD repair surgery reduces the rate of postoperative ERM formation (RR = 0.12, 95% CI 0.05–0.28).

Postoperative ERM formation is explained by migration of retinal pigment epithelium (RPE) cells into the vitreous through breaks in the neuroretina, which then induce myofibroblast transdifferentiation^24^. The internal limiting membrane (ILM) constitutes a scaffold for the development of proliferative membranes, as migrating and proliferating RPE and glial cells cause the formation of ERMs^25^. ILM peeling is used to treat different macular diseases, including macular holes, macular pucker (idiopathic ERM) and vitreoretinal traction syndrome, among others. In the case of preventing postoperative ERM formation, the beneficial effects of ILM peeling have been reported in several studies across the literature^4–13,26,27^. Eyes at increased risk of postoperative ERM are more likely to benefit from ILM peeling. Although some studies suggest certain features of RD such as macular involvement, retinal surface wrinkling, large or multiple retinal tears, vitreous haemorrhage and long-standing RD as risk factors for postoperative ERM development^5,6,8,11^, these findings are not consistent across studies, in relation to either the way they are reported or their statistical significance.

Our secondary meta-analyses also showed that ILM peeling during RD repair significantly lowered the need for secondary ERM surgery (RR = 0.05, 95% CI 0.02–0.17) and the rate of RD recurrence (RR = 0.51, 95% CI 0.28–0.94). In a cost-analysis of 6 studies, Yannuzzi et al.^19^ suggested that there are economic advantages associated with primary ILM peeling, even though the rate of secondary ILM peeling surgery is only reduced by 3–5%. ERM proliferation increases over time, and longer follow-up times may therefore provide further economic advantage^10^, despite most postoperative ERMs develop within 3 months after surgery^5^. Arias et al. classified postoperative ERM in three types according to the severity of the distortion caused to retinal layers, with a correspondingly increased likelihood of secondary ERM surgery^10^. RD recurrence may be prevented by ILM peeling, which theoretically eliminates retinal tension transmitted to the posterior pole by peripheral contraction if PVR develops^9^.

However, ILM peeling may also lead to complications, mainly related to mechanical trauma. These include vitreous, retinal or subretinal haemorrhage, retinal edema and retinal nerve fibre layer (RNFL) or photoreceptor damage, as well as eccentric scotoma, metamorphopsia and decreased light sensitivity^28^. In addition, ILM peeling in a detached retina represents a surgical challenge, as the mechanical avulsion of cellular proliferation on the retinal surface may alter the retinal microstructure^29^. Toxicity due to tamponade and staining agents has also been reported^30^.

The main functional result of postoperative ERM formation is a reduction in visual quality (in the form of metamorphopsia) and quantity. This meta-analysis showed that postoperative BCVA change did not differ significantly different between the non-ILM peeling and ILM-peeling groups (SMD = 0.14 logMAR; 95% CI -0.03–0.31). The results of the studies included are varied regarding this point, with some reporting better visual outcomes after ILM peeling, while others report worse outcomes. This variability may be explained by certain confounding factors within the study populations, including different follow-up time, preoperative macular status (on/off) and media opacities. Nam et al. reported that BCVA was better in the ILM-peeling group than in the non-ILM peeling group when only the macula-on group was analyzed^5^. Obata et al. show that BCVA did not differ significantly different between groups of macula-on patients. However, in eyes with macula-off RD, postoperative BCVA was significantly worse in the ILM peeling group than in the non-ILM peeling group^13^. As ILM peeling is technically more challenging in a detached macula than in a macula-on RD, greater damage may be caused in the first case. However, the underlying mechanism by which ILM peeling may hinder visual outcomes remains unclear. Further studies including a qualitative assessment (presence of metamorphopsia, scotomas, etc.) of vision are necessary.

This study has several limitations. First, the studies considered are all observational, and the only randomized clinical trial (RCT) screened was excluded because it involved patients with PVR grade C^3^. As subjects in the included studies were not randomized, more complicated cases may have been more likely in the ILM peeling group. Second, a qualitative approach to the assessment of visual acuity is missing in most studies, including microperimetry, visual fields and standardized subjective questionnaires. The lack of qualitative evaluation, together with the fact that all ERMs are usually considered in most studies, regardless of their severity, hinders making conclusions on the real benefit of ILM peeling being reached.

In summary, this systematic review and meta-analysis found that although ILM peeling during RD repair appears to reduce the rate of postoperative ERM, this advantage does not translate into consistent visual recovery across studies and the risk for potential complications associated with the intervention must be considered.

## Methods

### Data source and search methods

Two authors (DL and MB) searched PubMed for primary research papers published between January 2002 and August 2022 related to ILM peeling during primary RD repair. The following keywords and search strategy were used on September 9, 2022: ("internal limiting membrane" OR "ILM" OR "inner limiting membrane") AND "retinal detachment".

### Study selection

Clinical studies including RCTs, NRCSs and retrospective case series (n > 5 patients) related to ILM peeling during primary RD repair were included. Studies related to macular hole-associated retinal detachment, complex cases with PVR grade C or D, including only preoperative ERM, or performing only scleral buckling without vitrectomy, were excluded from this analysis. Articles in languages other than English or Spanish, literature reviews, comments on previous articles, case reports and small case series (n < 5 patients) were also excluded. This study adhered to the Preferred Reporting Items for Systematic reviews and Meta-analysis (PRISMA) guidelines^31^.

### Data extraction and outcomes

A web application (Rayyan)^32^ was used for screening titles and abstracts following the initial search. Two authors (DL and MB) independently reviewed the abstracts to ensure they met the eligibility criteria. The full-text versions of the eligible articles were assessed by both authors to select the studies for inclusion in this analysis. Discrepancies were resolved by discussion between the authors.

The following variables were extracted from the selected studies:

1. Study characteristics: year of publication, study type (RCT, prospective non-randomized study, retrospective case series), sample size, patient age, preoperative macula status (on/off), surgery description (including port gauge, technique, combined surgeries -cataract and/or scleral buckling- and stain used to assist ILM peeling) and minimum follow-up time.
2. Primary outcomes: rate of ERM formation following primary RD repair.
3. Secondary outcomes: mean best corrected visual acuity (BCVA) change after surgery (presented as mean with SD, in logMAR), rate of RD recurrence, need for secondary ERM surgery.

### Assessment of risk of bias and quality of studies

Risk of bias was assessed independently by two authors (DL and MB) using the Newcastle–Ottawa scale for non-randomized studies^33^, and any disagreement was resolved by discussion. The authors were contacted when relevant information was missing in the published manuscript. The Strengthening the Reporting of Observational Studies in Epidemiology Statement (STROBE) was used to assess the methodological quality of the studies^34.^ Publication bias was assessed graphically using funnel and Galbraith plots, as well as regression tests for funnel plot asymmetry.

### Data synthesis and analysis

The primary outcome measure was relative risk (RR) of postoperative ERM formation, with its corresponding 95% confidence interval (CI). The random effects model with restricted maximum likelihood estimation (Restricted Maximum Likelihood, REML) approach was used to analyze the data. The existence of statistical heterogeneity between studies was evaluated using the I^2^ index^35^, where values of over 40% were considered to represent moderate to considerable heterogeneity. *p*-values of < 0.10 were considered statistically significant. We expressed secondary outcomes as RR or standardised mean difference (SMD) with 95% CI and conducted random-effects model meta-analyses.

Subgroup analysis was also performed to assess the effect of certain covariables in the RR of postoperative ERM. These analyses were carried out by meta-regression with mixed model effects^36^.

The meta-analysis was carried out using the metafor package^37^, for the free software R (R Core Team, 2021).

## Supplementary Information

Supplementary Information 1.

Supplementary Information 2.

Supplementary Information 3.

## Data Availability

The data supporting the findings presented in this work are available from the corresponding author upon reasonable request.
