# Supplementary figures and images for: Primary ILM peeling during retinal detachment repair: a systematic review and meta-analysis
Source: Sci Rep. 2023 Mar 3;13:3586. doi: 10.1038/s41598-023-30060-w (PMC9984489; doi:10.1038/s41598-023-30060-w)

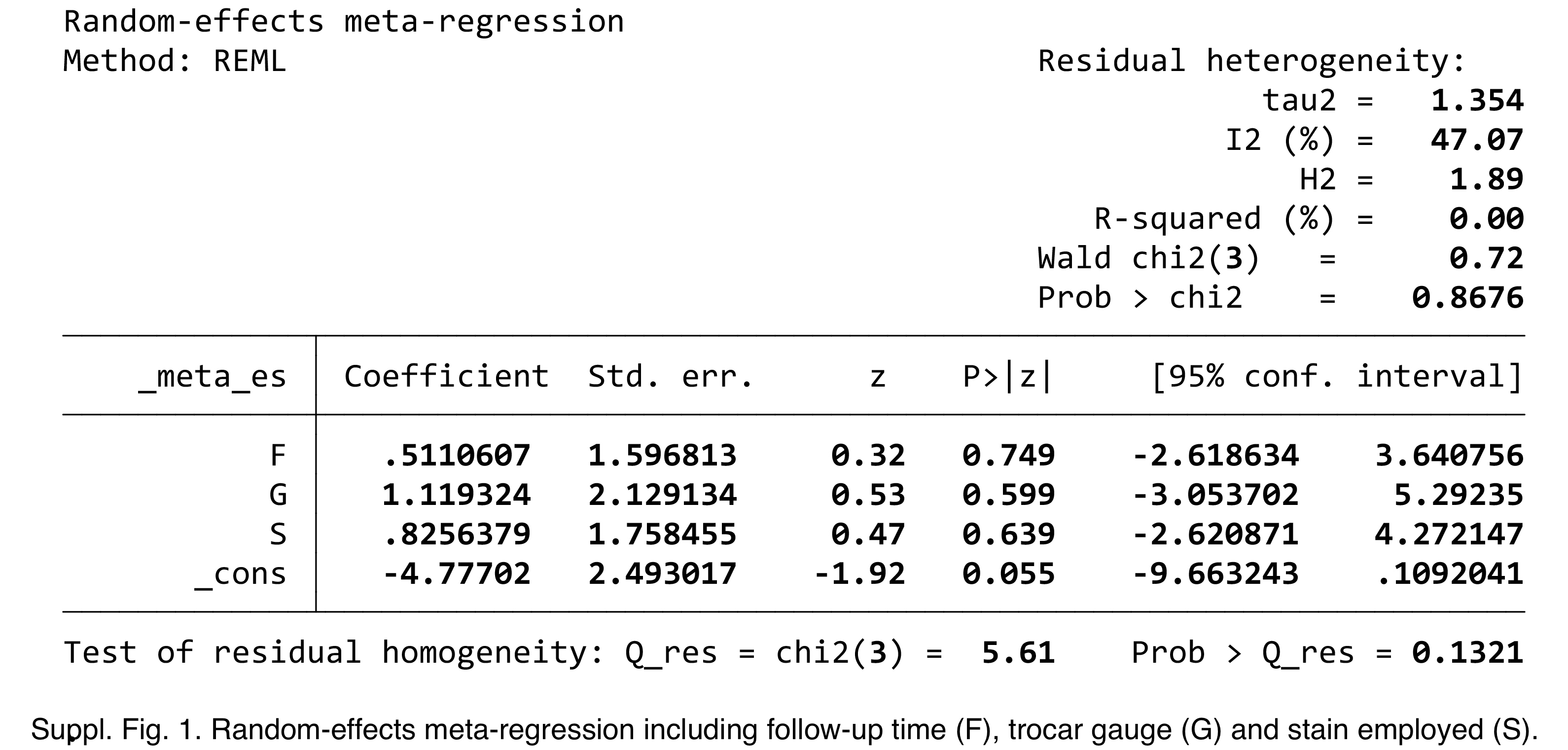

Supplement: Supplementary file 1 — Supplementary Information 1. [file 41598_2023_30060_MOESM1_ESM.jpeg]

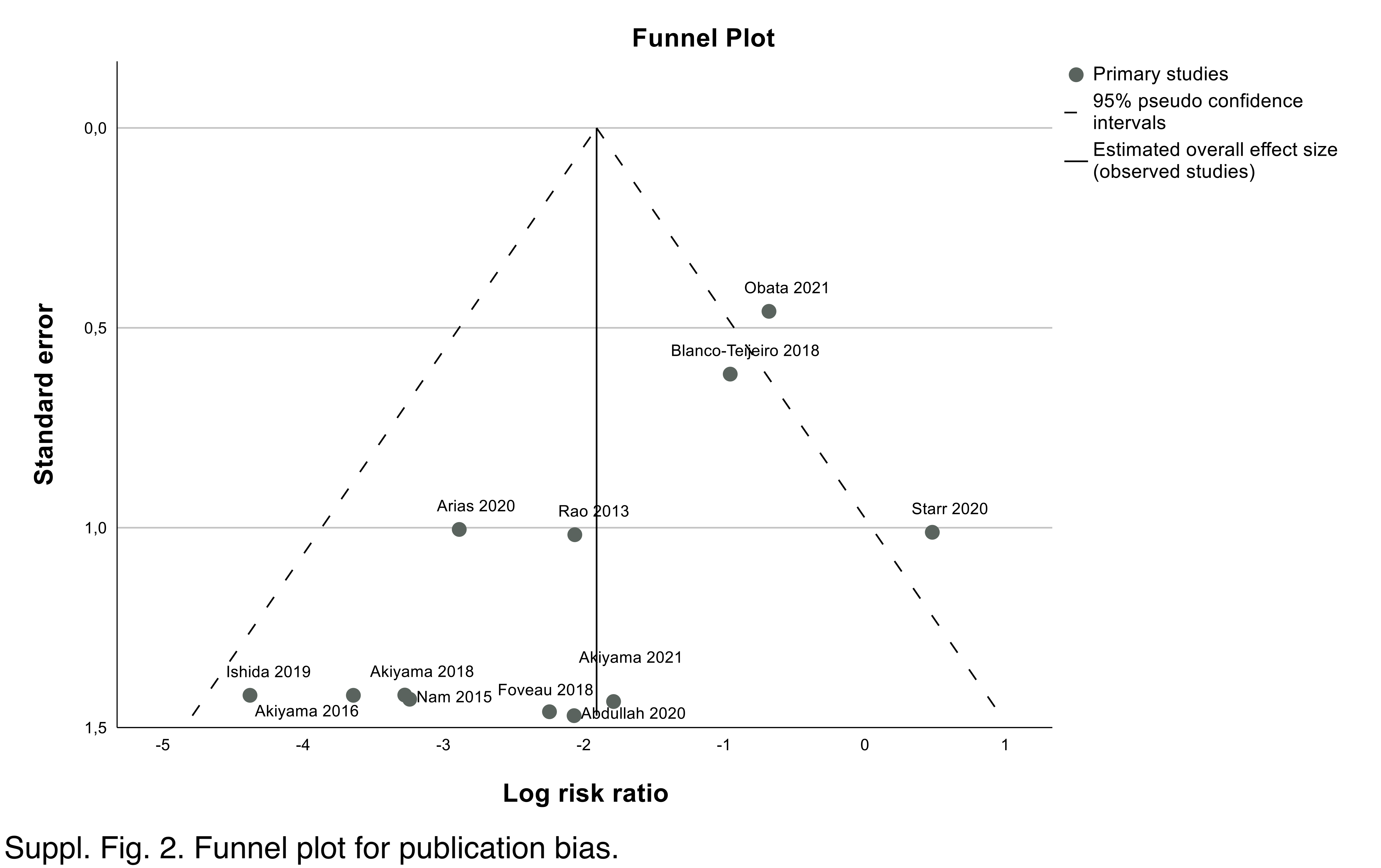

Supplement: Supplementary file 2 — Supplementary Information 2. [file 41598_2023_30060_MOESM2_ESM.jpg]

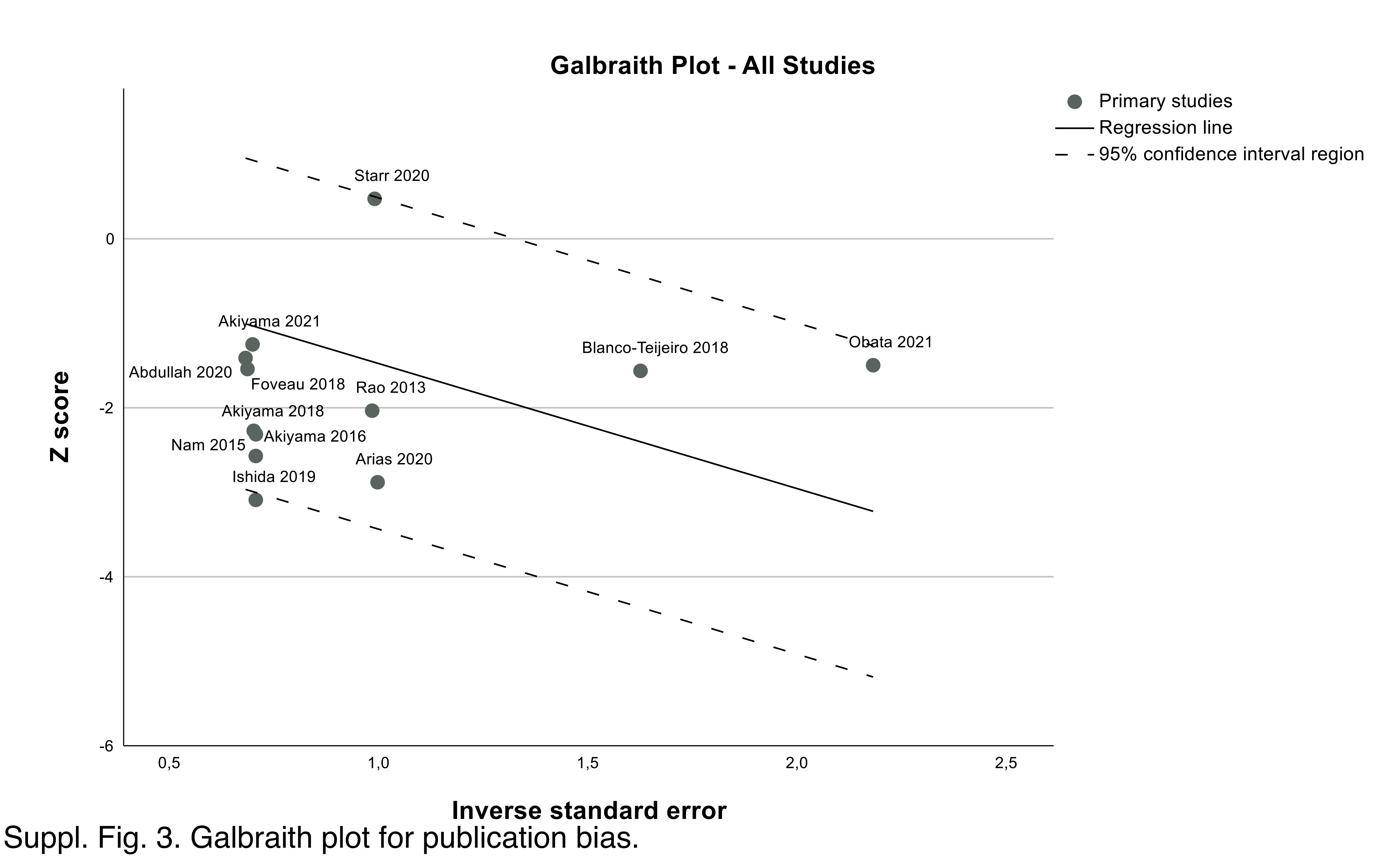

Supplement: Supplementary file 3 — Supplementary Information 3. [file 41598_2023_30060_MOESM3_ESM.jpg]
